# Performance analysis of indicators in teaching hospitals after the Health Transformation Plan: a Case Study in Iran

**DOI:** 10.1186/s41043-024-00642-z

**Published:** 2024-09-19

**Authors:** Bahman Ghasemzadeh, Mohammad Amerzadeh, Saeed Shahsavari, Saeideh Moosavi, Abdollah Keshavarz, Aisa Maleki, Rohollah Kalhor

**Affiliations:** 1 Student Research Committee, School of Public Health, Qazvin University of Medical Sciences, Qazvin, Iran; 2 Non-communicable Diseases Research Center, Research Institute for Prevention of Non-Communicable Diseases, Qazvin University of Medical Sciences, Qazvin, Iran; 3 Department of Epidemiology and Biostatistics, School of Health, Alborz University of Medical Sciences, Alborz, Iran; 4 Vice Chancellor of Treatment Affairs, Qazvin University of Medical Sciences, Qazvin, Iran; 5 Department of Health Management, Policy and Economics, School of Public Health, Tehran University of Medical Sciences, Tehran, Iran

**Keywords:** Performance indicators, Health Transformation Plan, Hospital, Iran

## Abstract

**Background:**

This study aimed to examine the status of performance indicators in hospitals affiliated with Qazvin University of Medical Sciences (QUMS) before and after the implementation of the Health Transformation Plan (HTP).

**Methods:**

This longitudinal descriptive-analytical study was conducted utilizing hospital data. The study collected data using a checklist that included both general characteristics of the participating hospitals and performance indicators such as “the number of outpatient visits,” “the number of paraclinical patients,” “the number of surgeries,” and “the number of inpatients” on a monthly basis for 2012–2019. The intervention examined in this study was the implementation of the HTP in May 2014. The data collected was analyzed using interrupted time series and STATA statistical software version 15.

**Results:**

The study examined seven hospitals affiliated with QUMS, including general, trauma, pediatric, gynecology, and psychiatry hospitals. The findings indicated a significant increase in outpatient visits, paraclinical patients, and inpatients in the first month after the intervention. Specifically, there was an increase of 1739 in the number of outpatient visits, an increase of 513 in the number of paraclinical patients, and an increase of 135 in the number of inpatients (*p* < 0.001).

**Conclusion:**

The HTP has improved patients’ access to medical services. It achieved this by reducing out-of-pocket payments for healthcare services and implementing programs such as developing clinics, improving the quality of visits, and retaining doctors in deprived areas. The reduction in out-of-pocket payments has been particularly beneficial for individuals who lack financial resources and previously faced barriers to accessing healthcare services.

## Background

Health is a fundamental capital for the development of societies. Healthy individuals are the driving force behind sustainable development [1]. The main objective of health systems is to enhance the overall health status of the community, protect against the high costs of healthcare services, and address the non-medical needs of the people [2, 3]. In recent years, the global approach to health has evolved significantly [4]. Advancements in knowledge and technology, coupled with an increase in public awareness, have led to a more comprehensive understanding of health and its various dimensions [5].

The factors influencing health and disease have undergone significant changes in recent times [6]. As a result, governments have taken major steps to ensure the accessibility of healthcare services by implementing reforms in their health systems [7, 8]. These reforms aim to improve the overall performance of health systems by changing various aspects of their functions [9]. To maintain the effectiveness of health systems, it is crucial to ensure that they are aligned with the global trend, which currently involves changes and reforms in most countries’ health systems worldwide [10, 11].

The Ministry of Health and Medical Education (MoHME) is responsible for overseeing the healthcare system in Iran. To achieve the objectives outlined in the vision of 2025 [12], which includes improving financial protection for the population, promoting equity in access to healthcare services, and enhancing the quality of services, the MoHME has implemented a reform program [1, 13, 14]. The reform program consists of seven programs focused on improving treatment services. These programs include “reducing patients’ payments in hospitals affiliated with the MoHME,” “improving the quality of visit services in hospitals affiliated with the MoHME,” “supporting the retention of physicians in deprived areas,” “enhancing the quality of accommodation services in hospitals affiliated with the MoHME,” “implementing a financial protection program for incurable, special, and needy patients,” “promoting natural childbirth,” and “implementing a specialist house physician program“ [1, 10, 13, 15].

Hospitals play a crucial role in providing healthcare services and contribute significantly to the government budget. Given their importance, it is essential to evaluate the quality of hospitals’ services [16]. Hospitals are responsible for providing prevention, early detection, timely treatment, and rehabilitation services to patients [17, 18]. The proper functioning of hospitals is critical in ensuring the recovery of patients and their return to society, and any mistakes can lead to significant consequences [19, 20].

The hospitals’ performance is crucial for improving the quality of life and has implications for other sectors, including social inequality, rising medical costs, and political problems [3]. Providing effective and efficient services requires the proper use of resources and improving productivity. Indicators are tools that can be used to monitor the hospitals’ performance, and accurate and continuous reporting of these indicators can improve their efficiency and effectiveness [21–23].

The World Health Organization (WHO) defines indicators as variables that can directly or indirectly measure change [24]. Therefore, evaluating changes and developments in the healthcare system requires using a set of indicators, including accessibility, financing, quality, and outcome indicators. Hospital performance indicators are one set of outcome indicators to measure changes in the health system performance indicators, such as the rate of inpatient admissions, outpatient visits, and surgeries are among the most critical indicators that should be regularly examined and evaluated to monitor the hospitals’ performance in different programs and periods [25–27]. The study aimed to investigate the status of performance indicators in hospitals affiliated with Qazvin University of Medical Sciences (QUMS) before and after the Health Transformation Plan (HTP) and to determine the impact of these reforms on the performance indicators of these hospitals.

## Methods

### Study design

This study was conducted longitudinally based on hospital data and utilized a descriptive-analytical approach. The research was carried out in all hospitals affiliated with QUMS. Data were collected by using a checklist that included general characteristics of hospitals, such as the type of hospital and their features, as well as indicators by month for the years between 2012 and 2019.

The hospitals’ performance was evaluated using four performance indicators approved and prioritized by the MoHME. The indicators used in the study were the number of outpatient visits, the number of paraclinical patients, the number of surgeries, and the number of inpatients. These indicators have been identified as the most important and widely used indicators for measuring the efficiency of hospitals and have been utilized in well-known models such as Pabon Lasso for measuring hospital performance [28–30].

### Performance indicators

#### The outpatient visits

It is a healthcare performance indicator that measures the proportion of patients who utilize the treatment and diagnostic services of a hospital’s outpatient department without occupying a hospital bed [31].

### The number of surgeries

It is a healthcare performance indicator that measures the number of surgeries performed in a hospital within a given period and about the number of operating room beds available in the hospital.

### The number of inpatients

An inpatient is an individual admitted to a hospital for examination, diagnosis, or treatment requiring at least one overnight stay. The inpatient admission rate refers to the number of admissions to hospital inpatient care per 1,000 people in a defined population, usually within a geographic region [31].

### Data collection

The data was collected from 2012 to 2019. The period consisted of two years before the HTP and five years after. The data was obtained from seven university hospitals in QUMS, Iran, relevant to specific months. BG visited the hospitals to collect the data with the assistance of statisticians, information officers, and informaticians from the respective hospital units. Subsequently, the collected data was verified by matching it separately with available data from the Treatment Vice Chancellor of the University and the Avab Health website of MoHME.^1^.

### Statistical analysis

The study employed an interrupted time series analysis using STATA version 15 statistical software. The data before and after the HTP were considered as time series. The plan’s effect on the level and trend of indicators after implementation was measured.

The interrupted time series model uses two variables to indicate the impact of an intervention: (1) the level variable which determines the immediate effect (2) the trend variable which shows the long-term impact. This means the immediate change in indicator levels at the start of the project (May 2014) and the monthly change thereafter were determined.

The stationarity of the data was checked using the Augmented Dickey-Fuller test to reject the null hypothesis of a unit root for all indicators, indicating stationary time series. The Chow test was used to determine structural breaks in the time series. Finally, serial autocorrelation in the regression residuals was determined to adjust the interrupted regression based on the degree of autocorrelation before estimating the model.

## Results

We reviewed seven hospitals, including three general hospitals, one trauma hospital, one pediatric hospital, one gynecology hospital, and one psychiatric hospital. The lowest and highest percentage of bed occupations were related to Amir Al-Momenin (31.2) and Bu Ali Sina (69.64) hospitals, respectively. The background information of the studied hospitals can be seen in Table 1.

**Table 1:** Background information of the studied hospitals (2019)

No.	Hospital	Geographical region	Type of hospital based on duty	Bed Turnover Rate (%)	Bed Occupancy Rate (%)	Average Length of Stay (Days)
1	Abu Ali Sina	Province’s center	General	3.523	69.648	6.402
2	Velayat	Province’s center	General	2.631	47.808	4.977
3	Shahid Rajaei	Province’s center	Trauma	8.331	69.616	2.557
4	Qods	Province’s center	Pediatric	3.009	36.410	3.642
5	Kosar	Province’s center	Obstetrics and Gynecology	3.738	51.279	2.750
6	22 Bahman	Province’s center	Psychology	1.674	86.888	16.234
7	Amir al-Momenin Buin Zahra	City	General	3.400	31.205	2.432
8	Rahimian	City	General	78.06	37.18	1.73
9	Takestan Tamin	City	General	83.24	53.06	2.34
10	Razi	Province’s center	General	67.95	53.78	2.91
11	Pasteur	Province’s center	General	142.76	62.62	1.72
12	Dehkhoda	Province’s center	General	93.12	62.37	3.05
13	Mehregan	Private	General	101.69	67.11	2.16
14	Vali-Asr Abyek	Private	General	58.53	45.2	1.9

(*p* < 0.001)

### The effect of health system reform on the number of outpatient visits

**Fig. 1:**
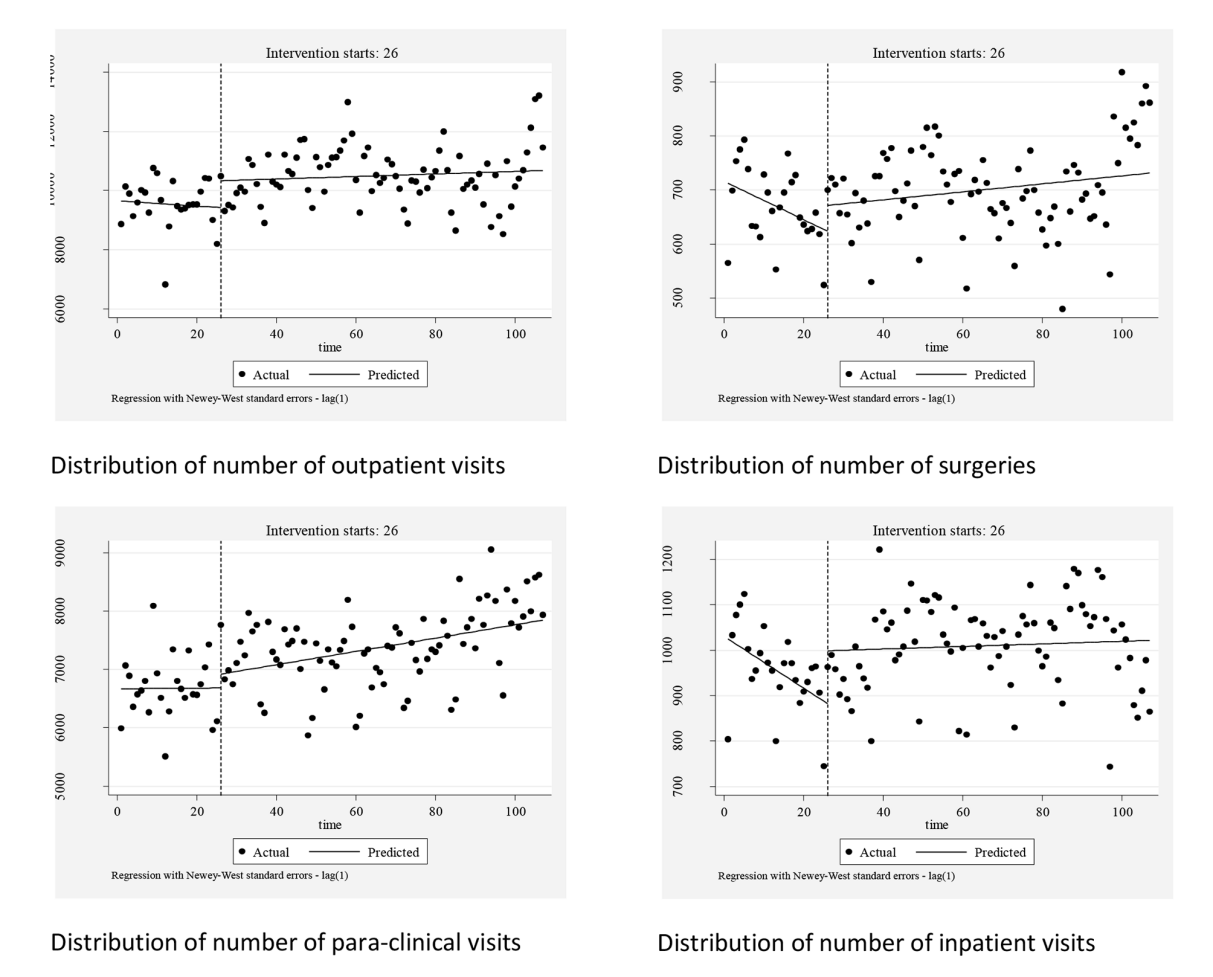
Distribution of various performance indicators among the investigated hospitals between April 2012 to May 2019

The interrupted regression results in Table 2 show that before the HTP, the initial number of outpatient visits was 6324, and the monthly changes in outpatient visits were significant for all hospitals (*p* < 0.001). In the first month after the intervention, outpatient visits increased significantly by 1739 (*p* < 0.001). Compared to the pre-intervention trend, outpatient visits increased by 106 per month post-intervention for all hospitals (*p* < 0.001). Figure 1 visually depicts the distribution of outpatient visits from April 2012 to May 2019.

### The effect of health system reform on the number of paraclinical patients

The interrupted regression results in Table 2 show that before the HTP, the initial number of paraclinical patients was 2846. The monthly changes in paraclinical patients’ pre-reform were insignificant for all hospitals. In the first month post-intervention, the number of paraclinical patients increased significantly by 513 (*p* < 0.001). Compared to the pre-intervention trend, paraclinical patients increased by 24 per month post-intervention across all hospitals (*p* < 0.001). Figure 1 visually depicts the distribution of paraclinical patients from April 2012 to May 2019.

### The effect of health system reform on the number of surgeries

The interrupted regression results in Table 2 show that before the HTP, the initial number of surgeries was 383. The monthly changes in the number of surgeries performed were insignificant for all hospitals. In the first month post-intervention, the change in the number of surgeries was also insignificant. Compared to the pre-intervention trend, the number of surgeries increased by 4.774 per month post-intervention across all hospitals (*p* = 0.001). Figure 1 visually depicts the distribution of the number of surgeries from April 2012 to May 2019.

### The effect of health system reform on the number of inpatients

The interrupted regression results in Table 2 show that before the HTP, the initial number of inpatients was 658. The monthly changes in the number of inpatients pre-reform were insignificant for all hospitals. In the first month post-intervention, the number of inpatients increased significantly by 135 (*p* < 0.001). Compared to the pre-intervention trend, the number of inpatients increased by 18 per month post-intervention across all hospitals (*p* < 0.001). Figure 1 visually depicts the distribution of the number of inpatients from April 2012 to May 2019.

**Table 2:** Estimation of interrupted time series model parameters for the effect of health system reform on the performance indicators

The number of outpatient visits							
	**Model coefficient**	**Standard deviation**	**t-statistic value**	**p-value**	**confidence interval 95%**		
					**Lower bound**	**Upper bound**	
y-intercept	6324.464	230.896	27.390	< 0.001	5866.536	6782.391	
Pre-intervention trend	-87.087	17.691	-4.920	< 0.001	-122.174	-52.000	
Level change after intervention	1739.270	385.631	4.510	< 0.001	974.461	2504.079	
Post-intervention trend change compared to pre-intervention	106.429	18.325	5.810	< 0.001	70.086	142.772	
Post-intervention trend	19.342	4.909	3.940	< 0.001	9.606	29.077	
(*p* < 0.001) The number of paraclinical patients							
	**Model coefficient**	**Standard deviation**	**t-statistic value**	**p-value**	**confidence interval 95%**		
					**Lower bound**	**Upper bound**	
y-intercept	2846.856	156.249	18.220	< 0.001	2536.973	3156.739	
Pre-intervention trend	-4.437	10.137	-0.440	0.663	-24.540	15.667	
Level change after intervention	513.987	166.672	3.080	0.003	183.432	844.542	
Post-intervention trend change compared to pre-intervention	24.498	10.883	2.250	0.027	2.914	46.082	
Post-intervention trend	20.061	3.108	6.455	< 0.001	13.898	26.225	
(*p* < 0.001) The number of surgeries							
	**Model coefficient**	**Standard deviation**	**t-statistic value**	**p-value**	**confidence interval 95%**		
					**Lower bound**	**Upper bound**	
y-intercept	383.349	24.611	15.580	< 0.001	334.539	432.158	
Pre-intervention trend	-2.258	1.320	-1.710	0.090	-4.876	0.361	
Level change after intervention	3.857	21.946	0.180	0.861	-39.667	47.382	
Post-intervention trend change compared to pre-intervention	4.774	1.395	3.420	0.001	2.008	7.541	
Post-intervention trend	2.516	0.461	5.460	< 0.001	1.602	3.431	
(*p* < 0.001) The number of inpatients							
	**Model coefficient**	**Standard deviation**	**t-statistic value**	**p-value**	**confidence interval 95%**		
					**Lower bound**	**Upper bound**	
y-intercept	658.853	37.076	17.770	< 0.001	585.322	732.383	
Pre-intervention trend	0.944	2.248	0.420	0.675	-3.515	5.403	
Level change after intervention	135.340	36.687	3.690	< 0.001	62.581	208.099	
Post-intervention trend change compared to pre-intervention	-0.400	2.396	-0.170	0.868	-5.152	4.353	
Post-intervention trend	0.544	0.782	0.696	0.488	-1.006	2.094	

(*p* < 0.001)

## Discussion

The study results indicate the HTP had positive impacts and changed most performance indicators in the hospitals before and after implementation. A comparison of the monthly average performance indicators before and after the HTP also showed significant changes. Similar studies have found that health system reforms can increase hospital workflow, evident in this study through higher inpatient volumes and bed turnover [31]. Several studies, including Sajjadi et al., Yousefzadeh et al., Rezaei et al., Dadgar et al., and Zarei et al., have reported similar findings regarding the impact of health system reform on hospital productivity and bed utilization. These studies have demonstrated that the HTP has had a significantly positive effect on these healthcare performance indicators [31–35].

The implementation of the HTP has had a significant positive impact on the people’s share of hospitalization costs in Iran, as reported by the MoHME. Before the HTP, the people’s share of hospitalization costs was 37%, but this decreased to 4.5% after the implementation of the reform. This has increased people’s access to health services, particularly for low-income groups, and has resulted in increased hospital admissions [32, 36, 37]. It is important to note that while the reduction of payments and the subsequent increase in performance indicators is a positive outcome of health system reform, it should not lead to an increase in induced demand among healthcare providers [37, 38].

The findings of Zarei et al.‘s study indicate a significant increase in outpatient visits, with a 26% increase reported [33]. The reasons for this increase are multifaceted. The development of special clinic programs and the plan to improve the quality of outpatient visits have led to a reduction in the payment of patients in outpatient departments. Additionally, the increase in the number of clinics and staff working hours has improved people’s access to health services. Similar findings have been reported in studies conducted by the WHO in different countries. These studies have shown that the implementation of interventions aimed at removing barriers to the use of health services, such as reducing patients’ payments and increasing accessibility, leads to an increase in the number of outpatient visits.

Rezaei et al. have confirmed the positive impact of the HTP on hospital performance indicators, including the bed occupancy rate, at Hamadan Hospital [39]. Similarly, Yaser et al.‘s study on the implementation of the health reform plan in the Turkish healthcare system found that it resulted in increased bed occupancy rates [40]. The employment rate in hospitals studied has also shown an increase of 10% after the implementation of the HTP. This increase can be attributed to several goals of the reform plan, such as reducing the amount of payment for patients, promoting the residency of physicians in deprived areas, and ensuring the presence of specialist doctors in hospitals affiliated with the MoHME. The plan also aimed to improve the quality of visiting and hospital hoteling services and provide financial protection for incurable patients [41].

The number of paraclinical patients in the current study has increased significantly after the HTP, which is contrary to the results of Farid Far et al.. The reason could be the number of years that have been investigated. In Farid Far et al’s study, only 2013 and 2014 have been considered, but in the present study, 2012 to 2019 are considered [42].

The present study has found a significant increase in the number of surgeries performed after the HTP. This finding suggests that the treatment of patients who required surgery was carried out promptly by doctors in the hospital. It is possible that the increase in the quality of visiting and hoteling services of the hospital after the HTP contributed to this increase in the number of surgeries. These results are consistent with Rezaei et al. and Dadger et al. who found a positive impact of the HTP on the number of surgeries performed in hospitals [39, 43]. Similarly, a study conducted in Turkey reported a significant increase in the number of surgeries after the implementation of the health reform plan [44].

This study has several limitations. One limitation is the lack of control over other influential and confounding factors that may have impacted the study’s performance indicators. Therefore, the changes observed in the indicators cannot be definitively attributed to the HTP alone. However, it is important to note that no other major interventions were implemented during the HTP that could have influenced the results. Additionally, this study only examined three widely used performance indicators, and other important indicators such as accessibility and justice, quality and effectiveness (such as readmission rate, nosocomial infection rates, staff and patient satisfaction, hospital complaints, and the rate of medical errors) were not analyzed. Future studies should consider analyzing these additional indicators to provide a more comprehensive evaluation of the impact of the HTP on the healthcare system.

## Conclusion

The results of this study demonstrate that the HTP had a positive impact on hospital performance indicators. The reform increased access to medical services for patients by reducing out-of-pocket payments and implementing programs, such as developing clinics and improving the quality of visits. The availability of physicians in deprived areas has also improved, which is particularly beneficial for people who have not pursued medical care due to financial constraints. The positive effects of the HTP highlight the importance of implementing policies and programs that promote universal health coverage and access to healthcare services.

## Data Availability

The datasets used and/or analyzed during the current study available from the corresponding author on reasonable request. The entire dataset is in Farsi language. The Data can be available in English language for the readers and make available from the corresponding author on reasonable request..

## Footnotes

1 Avab.behdasht.gov.ir.
